# Supplementary material for: Does increasing social presence enhance the effectiveness of writing explanations?
Source: PLoS One. 2021 Apr 22;16(4):e0250406. doi: 10.1371/journal.pone.0250406 (PMC8062030; doi:10.1371/journal.pone.0250406)
Supplement: Attachment — Submitted filename: Response to Reviewers.docx [file pone.0250406.s002.docx]

Dear Dr. Castro-Alonso,

Dear reviewers,

Thank you very much for your editorial efforts and the opportunity to resubmit our manuscript. Hereby, we would like to submit our revision in which we addressed all the issues raised by the reviewers. We carefully revised our manuscript accordingly and responded to each comment with utmost care. For readability, the reviewers’ comments are printed in bold and responses in regular font. Furthermore, we additionally highlighted all changes in the manuscript in quotation marks in the rebuttal letter.

We are convinced that the reviewers’ comments helped us improve our work and hope that we revised the manuscript as expected.

Yours sincerely,

The Authors

**Reviewer #1**

**Dear authors,

thank you for this thorough and overall impressive revision. I think the additions you made in this revision add to the overall quality of the manuscript and I feel that my comments have been adequately addressed. In total, I think this is important and rigorous work and I'm happy to recommend acceptance.**
Thank you very much for your appreciative words and your work which helped us improving our manuscript.

**Reviewer #2**

**The authors addressed all concerns raised by the reviewers in their revision. I have only some minor comments.**

We addressed all comments with utmost care.

**Page 8
The results of previous studies are described in too much detail. I think that details of the types of statistical analyses applied are not relevant or even not easily comprehensible (e.g., contrast analyses, serial mediation analyses).**

We revised the corresponding paragraph accordingly by avoiding specific analyses and reformulated sentences to facilitate the reading process (p. 7-8).

“Results revealed an interaction effect between learning activity and text difficulty (*d* = 0.31). The effect of explaining was only significant in the high-difficult condition, but not in the low-difficult condition. Thus, the explaining effect only held true when students learned from difficult but not from less difficult material. More interestingly, students who explained the contents to a fictitious peer orally outperformed students who wrote an explanation. Again, this effect was only significant when the learning material was difficult, but not when it was less complex. Additionally, perceived social presence and the richness of explanations mediated the explanatory effect: Students who explained orally perceived a stronger presence of the fictitious peer (measured by the number of personal references) compared to students who wrote an explanation. Higher levels of social presence, in turn, was associated with richer explanations (measured by the number of mentioned concepts) which resulted in better learning outcomes, at least for the difficult text.”

**Page 12
The reliability of the knowledge tests is relatively low. Please explain why. In this context, please explain why you used McDonald’s reliability index.**
We used the knowledge tests for measuring prior-knowledge and comprehension referring to different non-necessarily related sub-topics to cover students’ comprehensive understanding. In other words, we measured a broad knowledge base of the topic “immunology” by asking different and independent questions regarding different subtopics of immunology. This pattern of questions will necessarily result in low internal consistency (Tavakol & Dennick, 2011), as there is a trade-off between comprehensiveness and consistency. Therefore, we would argue from a theoretical perspective that low consistencies say little about the quality of the measurement (see also Scheiter et al., 2015; Stadler et al., 2021). Nevertheless, we discussed the low reliability values in the limitation section on pages 28-29 in the revised manuscript.

“Another limitation refers to the relatively low homogeneity indices. However, we used validated tests which were implemented in prior studies. Additionally, we want to note that we measured a broad understanding of the topic by asking different and independent questions with a restricted set of 5 to 6 items per test. This decision likely resulted in a trade-off regarding the internal consistency of the knowledge measures, as they likely covered diverse sub-components of students’ comprehension [1]. Further research with advanced statistical procedures is needed which explicitly takes the multidimensionality into account, for instance, by using bifactor-(S-1) measurement models which, however, require larger sample sizes [2].”

Regarding the homogeneity indices, we want to note that using Cronbach’s alpha is discussed as rather problematic for determining the reliability of knowledge tests. For instance, Cronbach’s alpha is commonly regarded as appropriate measure for the internal consistency of one single construct (Dunn et al, 2014) but not of multidimensional construct as in the present study. McDonald’s Omega, in contrast, is an adequate measure for reliability for multidimensional constructs (Hayes & Coutts, 2020). Besides the revised discussion paragraph, we additionally highlighted the multidimensionality of our constructs on page 12 in the revised manuscript:

“We used the prior knowledge test from Golke and Wittwer [3], which contained five questions with an open-ended answer format and represented a multidimensional construct, measuring different subcomponents of immunology (e.g., “Why can viruses be dangerous for humans?”; McDonald’s ω_t_ = .60). […] We measured students’ text-based knowledge and inference knowledge with two different posttests from Golke and Wittwer [3]. The tests consisted of different questions than the prior knowledge test and represented multidimensional constructs.”

Dunn, T. J., Baguley, T., & Brunsden, V. (2014). From alpha to omega: A practical solution to the pervasive problem of internal consistency estimation. *British Journal of Psychology*, 105, 399-412.

Hayes, F. A., & Coutts, J. J. (2020). Use omega rather than cronbach’s alpha for estimating reliability. But…. *Communication Methods and Measures*, 14(1), 1-24.

Scheiter, K., Schüler, A., Gerjets, P., Huk, T., & Hesse, F. W. (2015). Extending multimedia research: How do prerequisite knowledge and reading comprehension affect learning from text and pictures. *Computer in Human Behavior*, 31, 73-84.

Stadler, M., Sailer, M., & Fischer, F. (2021). Knowledge as a formative construct: A good alpha is not always better. *New Ideas in Psychology*, 60, 100832.

Tavakol, M., & Dennick, R. (2011). Making sense of Cronbach's alpha. *International Journal of Medical Education*, 2, 53-55.

**Page 14
The authors provide a rho coefficient to indicate reliability of the tests. Please explain what this coefficient means.**

We calculated Spearman Brown’s rho for enjoyment and interest as both scales contain only two items and rho presents a correlative measure for reliability. However, in terms of cohesion, we now calculated McDonalds Omega (p. 14).

“We measured students’ enjoyment during the learning activity by using two self-generated items (e.g., “I enjoyed doing the task”). The students rated their enjoyment on a 4-point Likert scale from 1 “not at all” to 4 “absolutely”. Reliability was good (McDonald’s ω_t_ = .96). […] We measured students’ enjoyment during the learning activity by using two self-generated items (e.g., “I enjoyed doing the task”). The students rated their interest on a 4-point Likert scale from 1 “not at all” to 4 “absolutely”. Again, reliability was good (McDonald’s ω_t_ = .81).”

**Discussion
The authors found that all students overestimated their performance in the knowledge test. However, the authors do not explain why students did so. Please explain.**

Prior research demonstrated that students generally overestimate their current understanding (Eitel, 2016; Mayer et al., 2007; Prinz et al., 2020). Thus, we did not attribute this finding to our study but rather to students’ tendency of overestimating their comprehension. We discussed the finding in our discussion section on page 25 in the revised manuscript.

“Additionally, students showed comparable monitoring accuracy ratings across conditions. Interestingly, all students overestimated their current understanding. This, however, is in line with prior research that highlighted that students generally tend to overestimate their current comprehension [4–6]. Nevertheless, we need to reject our hypotheses regarding effects on students’ comprehension and their monitoring accuracy.”

**I think another limitation of the authors’ study is that students who were not familiar with the topic to be explained served as participants. Given that explaining is usually a task that is not done routinely (Rozenblit & Keil, 2002), I wondered whether these students were generally interested in explaining an unfamiliar topic. Maybe, this fact might also explain the null results for learning.**

**Rozenblit, L., & Keil, F. (2002). The misunderstood limits of folk science: An illusion of explanatory depth. Cognitive Science, 26(5), 521–562.** [**https://doi.org/10.1207/s15516709cog2605_1**](https://doi.org/10.1207/s15516709cog2605_1)

Explaining new and unfamiliar contents is a common learning activity in research on generative learning activities (Brod, 2020; Fiorella & Mayer, 2016). Thus, our approach did not differ from prior research (see Fiorella & Mayer, 2013, 2014; Fukaya, 2013; Hoogerheide et al., 2016, 2019; Lachner et al., 2020, 2021). Nevertheless, our null findings might be due to low levels of prior knowledge. However, prior research revealed that generating an explanation is particularly beneficial for students with low prior knowledge (see Hoogerheide et al., 2019). We discussed students’ low prior knowledge in relation to the null findings in the discussion section on page 25 in the revised manuscript.

“The null findings regarding students’ comprehension might be attributed to low levels of prior knowledge. Little prior knowledge limits students in learning new contents adequately [7, 8]. However, prior research revealed that generative learning activities, such as explaining, are in particular beneficial for low prior knowledge students [9].”

We discussed the challenge of generating an explanation in the discussion section on page 27 in the revised manuscript.

“Nevertheless, against the background of the relatively low quality of students’ explanations, as a theoretical consequence of our study, we suggest that future research should focus on how students could be supported to generate high-quality explanations. Given that students are generally not familiar with explaining challenging contents [10], they might depend on further support to be able to apply this learning activity successfully. As a first attempt, Lachner and Neuburg [11] investigated the role of formative feedback during explaining (in written form). They found that formative feedback helped students generate more cohesive explanations, which finally contributed to their comprehension. Additionally, the self-explaining literature focusses on two main instructional approaches [12], supporting self-explaining directly by means of pre-trainings [13] or indirectly by the use of prompts [14, 15], which should work as strategy activators to enact deep-level explaining strategies. Whether and under which conditions such direct and indirect support procedures also hold true for generating explanations to fictitious others has to be investigated by further research.”

Brod, G. (2020). Generative learning: Which strategies for what age? *Educational Psychology Review.* Advance online publication. https://doi.org/10.1007/s10648-020-09571-9

Fiorella, L., & Mayer, R. E. (2013). The relative benefits of learning by teaching and teaching expectancy. *Contemporary Educational Psychology*, *38*(4), 281–288. https://doi.org/10.1016/j.cedpsych.2013.06.001

Fiorella, L., & Mayer, R. E. (2014). Role of expectations and explanations in learning by teaching. *Contemporary Educational Psychology*, *39*(2), 75–85. https://doi.org/10.1016/j.cedpsych.2014.01.001

Fiorella, L., & Mayer, R. E. (2016). Eight ways to promote generative learning. *Educational Psychology Review*, *28*(4), 717–741. https://doi.org/10.1007/s10648-015-9348-9

Fukaya, T. (2013). Explanation generation, not explanation expectancy, improves metacomprehension accuracy. *Metacognition Learning*, *8*(1), 1–18. https://doi.org/10.1007/s11409-012-9093-0

Hoogerheide, V., Deijkers, L., Loyens, S. M., Heijltjes, A., & van Gog, T. (2016). Gaining from explaining: Learning improves from explaining to fictitious others on video, not from writing to them. *Contemporary Educational Psychology*, *44-45*, 95–106. https://doi.org/10.1016/j.cedpsych.2016.02.005

Hoogerheide, V., Renkl, A., Fiorella, L., Paas, F., & van Gog, T. (2019). Enhancing example-based learning: Teaching on video increases arousal and improves problem-solving performance. *Journal of Educational Psychology*, *111*(1), 45–56. https://doi.org/10.1037/edu0000272

Lachner, A., Backfisch, I., Hoogerheide, V., van Gog, T., & Renkl, A. (2020). Timing matters! Explaining between study phases enhances students’ learning. *Journal of Educational Psychology*, *112*(4), 841–853. https://doi.org/10.1037/edu0000396

Lachner, A., Jacob, L., & Hoogerheide, V. (2021). Learning by writing explanations: Is explaining to a fictitious student more effective than self-explaining? *Learning and Instruction*.

**References**

1. Dunn TJ, Baguley T, Brunsden V. From alpha to omega: A practical solution to the pervasive problem of internal consistency estimation. British Journal of Psychology 2014; 105(3):399–412.

2. Eid M, Geiser C, Koch T, Heene M. Anomalous results in G-factor models: Explanations and alternatives. Psychol Methods 2017; 22(3):541–62.

3. Golke S, Wittwer J. High-performing readers underestimate their text comprehension: Artifact or psychological reality? Proceedings of the 39th Annual Conference of the Cognitive Science 2018.

4. Eitel A. How repeated studying and testing affects multimedia learning: Evidence for adaptation to task demands. Learning and Instruction 2016; 41:70–84.

5. Mayer RE, Stull AT, Campbell J, Almeroth K, Bimber B, Chun D et al. Overestimation bias in self-reported SAT scores. Educational Psychology Review 2007; 19(4):443–54.

6. Prinz A, Golke S, Wittwer J. To What Extent Do Situation-Model-Approach Interventions Improve Relative Metacomprehension Accuracy? Meta-Analytic Insights. Educ Psychol Rev 2020; 32(4):917–49.

7. Brod G. Generative learning: Which strategies for what age? Educational Psychology Review 2020.

8. Renkl A. Lehren und Lernen. In: Tippelt R, Schmidt-Hertha B, editors. Handbuch Bildungsforschung. Wiesbaden: Verlag für Sozialwissenschaften; 2009. p. 737–51.

9. Hoogerheide V, Renkl A, Fiorella L, Paas F, van Gog T. Enhancing example-based learning: Teaching on video increases arousal and improves problem-solving performance. Journal of Educational Psychology 2019; 111(1):45–56.

10. Rozenblit L, Keil F. The misunderstood limits of folk science: An illusion of explanatory depth. Cogn Sci 2002; 26(5):521–62.

11. Lachner A, Neuburg C. Learning by writing explanations: Computer-based feedback about the explanatory cohesion enhances students’ transfer. Instructional Science 2019; 47(1):19–37.

12. Fonseca BA, Chi MTH. Instruction based on self-explanation. In: Mayer RE, Alexander PA, editors. Handbook of research on learning and instruction. New York: Routledge; 2011 (Educational psychology handbook series).

13. McNamara DS. Self-explanation and reading strategy training (SERT) improves low-knowledge students’ science course performance. Discourse Processes 2017; 54(7):479–92.

14. Chi MTH, Leeuw N de, Chiu M-H, LaVancher C. Eliciting self-explanations improves understanding. Cogn Sci 1994; 18(3):439–77.

15. Schworm S, Renkl A. Learning argumentation skills through the use of prompts for self-explaining examples. Journal of Educational Psychology 2007; 99(2):285–96.
